# Reducing catheter-associated urinary tract infections in a large health system: a quality improvement approach using a fractal management system

**DOI:** 10.1017/ash.2024.386

**Published:** 2024-09-25

**Authors:** Elie A. Saade, Esther J. Thatcher, Tina Lewis, Susan Carr, Marcia Cornell, Rachel Arnold, Zainab Albar, Peter Pronovost

**Affiliations:** 1 Case Western Reserve University School of Medicine, Cleveland, OH, USA; 2 Division of Infectious Diseases and HIV Medicine, Department of Internal Medicine, University Hospitals, Cleveland, OH, USA; 3 Quality Institute, University Hospitals, Cleveland, OH, USA; 4 Population Health, University Hospitals, Cleveland, OH, USA; 5 Geauga Medical Center, University Hospitals, Chardon, OH, USA; 6 Kent State University College of Nursing, Kent, OH, USA; 7 Chief Quality and Clinical Transformation, University Hospitals, Cleveland, OH, USA; 8 Frances Payne Bolton School of Nursing and Weatherhead School of Management, Case Western Reserve University, Cleveland, OH, USA

## Abstract

**Objective:**

Although preventable through established infection control practices, catheter-associated urinary tract infections (CAUTIs) remain prevalent in acute-care settings. Our goal was to improve the CAUTI rates through multiple hospitals through implementing sustainable practices, including enhancing communication, provider engagement, accountability, and transparency in reporting to achieve long-term improvements.

**Design:**

Quality improvement with multiple levels of interventions

**Setting:**

A health system in northern Ohio with 21 affiliated hospitals across 16 counties.

**Patients:**

Adult patients admitted to the hospital between June 2020 and June 2023.

**Methods:**

A broad set of quality improvement (QI) strategies was developed by an interdisciplinary team and guided by the Fractal Management System framework to ensure accountability, communication, and alignment across teams and facilities. Key drivers were indwelling urinary catheter (IUC) alternatives, insertion, maintenance, removal, and smart diagnostics. The main outcome measures were standardized infection ratios (SIR) and standardized utilization ratio (SUR), comparing period 1 (P1, June 2020 to December 2021) and period 2 (P2, January 2022 to June 2023).

**Results:**

Enhanced communication and management played crucial roles in minimizing IUC placement. Updated policies and protocols, coupled with clear guidelines and decision support tools, facilitated effective urinary management. Performance tracking and visual management boards provided real-time insights, while collaborative efforts, including staff huddles and multidisciplinary teamwork, ensured consistent adherence to best practices.

**Conclusions:**

A systemwide QI initiative focused on enhanced communication, management, and collaboration contributed to improved SIR and reduced CAUTI rates across multiple hospitals, highlighting the impact of strong communication and proactive management in healthcare settings.

## Introduction

Catheter-associated urinary tract infections (CAUTIs) remain among the most common healthcare-associated infections (HAIs), with an estimated incidence of 0.2% among hospitalized patients in acute-care hospitals in 2015, representing nearly 10% of all HAIs.^1^ CAUTI rates have not reached the national goal of a “never event”; they decreased up until the beginning of the coronavirus disease 2019 pandemic and then increased again.^2,3^ Most CAUTIs are preventable through rigorous infection control practices, including strict hand hygiene, aseptic insertion using sterile supplies, and timely removal of urinary catheters.^3^ However, implementing these multicomponent improvements at scale across complex health systems is challenging.

The existing literature underscores the critical importance of employing multidisciplinary and multifaceted methods that integrate various sustainable, evidence-based strategies alongside effective education and feedback to achieve lasting improvements in patient care quality, particularly in reducing CAUTIs across multiple healthcare units or facilities. For instance, Reynolds et al evaluated a multifaceted intervention to reduce CAUTIs in three intensive care units (ICUs), which included cognitive aids, printed educational materials, educational outreach visits, and real-time feedback to ICU staff. This intervention led to sustained reductions in urine culture rates, catheter utilization, and CAUTI incidence over a 4-year postintervention period, highlighting the effectiveness and sustainability of evidence-based strategies in infection prevention and control.^4^ Similarly, Mullin et al implemented a multifaceted intervention in ICUs that emphasized the “stewardship of culturing” to align culturing practices with established guidelines. Their interventions, which included assessing competency with catheter insertion and maintenance, maintaining a closed system, initiating a nursing-driven catheter removal protocol, improving electronic documentation, using preservative tubes for specimen collection, and conducting periodic maintenance audits, collectively resulted in a significant reduction in CAUTI rates.^5^

At University Hospitals of Cleveland (UH), a multifacility healthcare system in northern Ohio, the increasing frequency of CAUTIs prompted administrators to refocus on quality improvement (QI) initiatives aimed at preventing this complication. To address this issue, the Quality Institute established an adaptable infrastructure centered on setting precise objectives, granting autonomy, fostering flexibility, and facilitating communication between frontline teams. This approach, referred to as the “Fractal Management System,” has been successfully applied in various healthcare settings, including primary care and patient safety.^6–8^ This report details the application of this method to infection prevention practices, demonstrating its potential to enhance quality across diverse healthcare environments.

## Methods

The FMS framework guided the development and deployment of a broad set of QI strategies to reduce CAUTI rates (Table 1). A matrix of accountability and communication was established to identify various teams within each facility as well as at the broader health system level, with the intent of aligning their efforts.

**Table 1.** Fractal management system framework for the CAUTI project

Pillar	Action	CAUTI Project Example
Declare goals	Create goals	CAUTI SIR is lower than the 70^th^ percentile of the national benchmark for the most recent reporting year.
	Establish roles	Central system-level and facility-level interdisciplinary committees, including DRI.
	Set expectations	Setting the expectation for providers and staff to maintain accurate documentation and timely performance of tasks.
	Align incentives	Instituting rewards or recognition for teams that reach set performance targets or display significant improvement.
Create enabling infrastructure	Define and measure goals and key behaviors	Tracking the standardized utilization ratios of indwelling urinary catheters, frequency of completion of chlorhexidine baths and showers, and proportion of placement and maintenance orders present.
	Create real-time data dashboards.	Nursing unit-level dashboards to monitor the presence of indwelling urinary catheters and completion of chlorhexidine baths and showers.
	Establish project management support	A dedicated project management team or professional coordinates and monitors the project’s progress and communicates updates.
	Develop playbook	Playbook shared across teams with promising practices and lessons learned.
	Create scorecards for retrospective analytics	Created scorecards that reflect the progress toward the completion of outcome and process goals.
	Create a communication plan	Policies, trainings, one-pagers, email newsletters, one-on-one conversations, and regular feedback on performance.
Engage and connect	Create a fractal management structure connecting the board to the bedside	A multidisciplinary core team guides and supervises the work. Individual hospital facilities form their operational groups.
	Implement peer learning groups	Horizontal structures allow for peer learning and sharing of best practices among hospitals and units.
	Service line level	Establishing service line-level connections to focus on process and objective goals.
	Site level	Fostering site-level engagement among clinical and practice leaders.
Report transparently and ensure shared accountability	Define accountability structure	Defining an accountability structure in the form of a Responsible, Accountable, Consulted, Informed (RACI) matrix.
	Hold regular accountability meetings	Updated outcome and process measures reviewed during facility and unit-level meetings and regularly reported to the central core.
	Distribute data scorecards	Creation and distribution of monthly scorecards to all team members showing progress toward the goals.

Note. CAUTI, catheter-associated urinary tract infection; SIR, standardized infection ratio; DRI, directly responsible individual.

### The centralized core team and its roles

A centralized, multidisciplinary team oversaw the work, comprising executives, QI specialists, infection preventionists, supply chain experts, nursing and medical content experts, education specialists, data analysts, and project managers. Each facility had a directly responsible individual (DRI) who provided updates during system-level meetings, while additional representatives shared insights, updates, and lessons learned.

### Operational groups at each facility

Every hospital facility formed its own operational group led by the DRIs, emulating the structural blueprint at the system level. These groups utilized professionals with similar roles and expertise, who were attuned to the culture and functioning of their respective facilities. This enabled groups to adapt and replicate initiatives developed by the central core team, organize and train bedside patient-adjacent teams, provide supervision, and ensure accountability.

### Goal-setting and accountability

**Figure 1.**
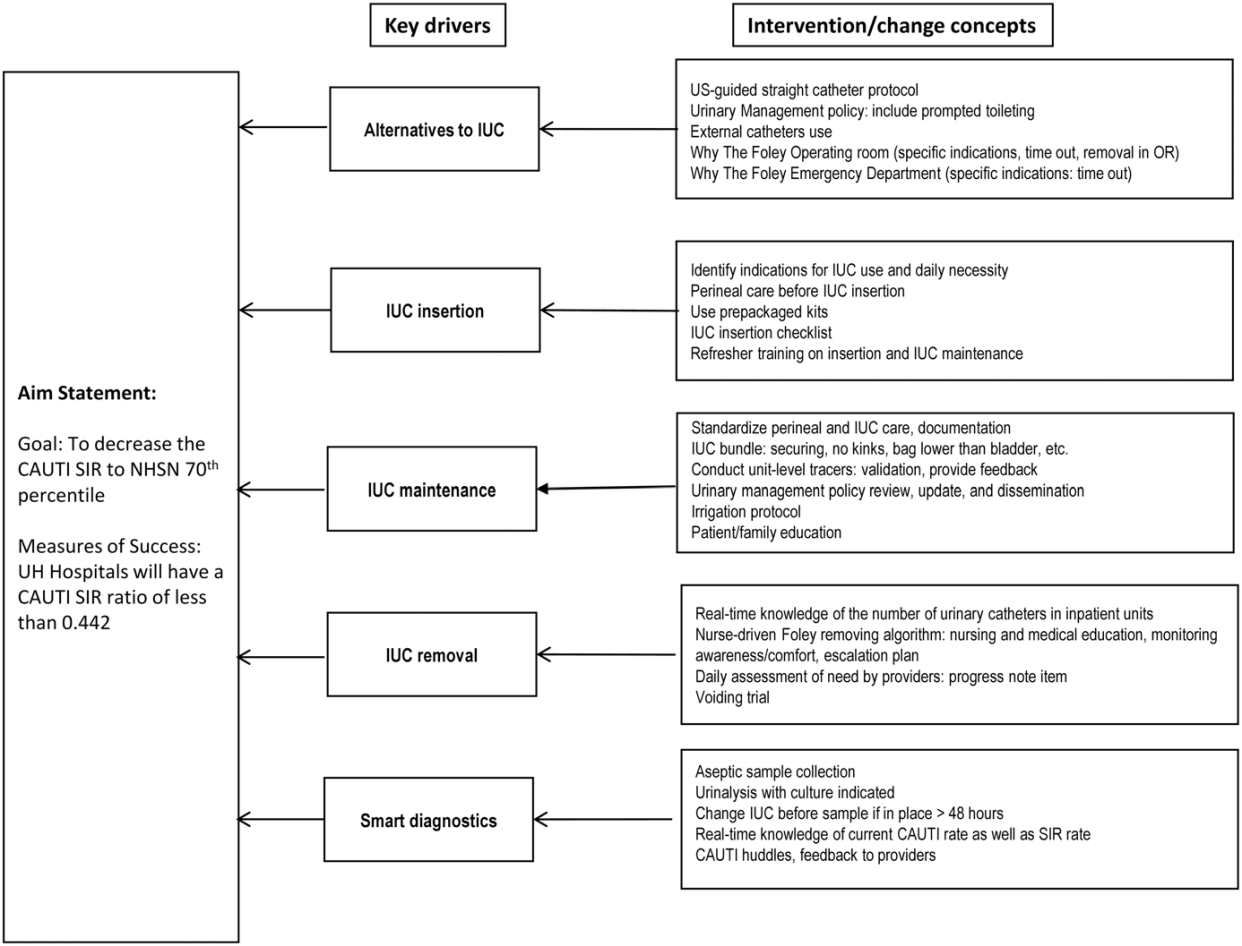
Key driver diagram for CAUTI quality improvement project. CAUTI, catheter-associated urinary tract infection; SIR, standardized infection ratio; IUC, indwelling urinary catheter; US, ultrasound; OR, operating room.

The Key Driver Diagram provides a visual roadmap of the objectives and systematic efforts in the FMS to achieve the targeted improvement aim (Figure 1). The key drivers to reduce CAUTI included alternatives to indwelling urinary catheter (IUC) usage, proper IUC insertion and maintenance, effective IUC removal, and smart diagnostics. The corresponding interventions included an ultrasound-guided straight catheter protocol and proper perineal care before IUC insertion as well as daily IUC necessity assessment and real-time CAUTI rate monitoring. Goals for these interventions were outlined using the Specific, Measurable, Achievable, Relevant, and Time-bound (SMART) framework.^9^ For instance, goals included a 90% compliance rate for daily chlorhexidine bathing and 95% compliance in documentation of catheter necessity. These objectives and transparent measures were tracked at each facility level and throughout the organization.

### Horizontal and vertical communication structures

Consistent communication of the initiative’s goals, objectives, initiatives, and interventions throughout a complex network of vertically and horizontally aligned teams was the core of achieving the desired systemwide outcome. The communication goal was for everyone at every level of the organization (bedside to board room) to understand and communicate the initiative’s objectives and interventions.

Horizontal structures encourage peer-to-peer discussions and team collaboration, which are crucial for spreading best practices across different hospitals and units. They provided a space for sharing strategies, tackling challenges, and creating solutions. These structures allowed facilities to learn from others who had already faced and overcome similar challenges. For example, an intensive care unit with minimal IUC usage pioneered an algorithm to guide an ultrasound-based intermittent bladder catheterization protocol. This protocol was eventually scaled up as a comprehensive system guideline.

Vertical communication structures within the FMS encouraged accountability, stimulated the spread of innovative solutions, and promoted shared problem-solving and learning. In the context of the CAUTI prevention project, these vertical links connected clinical units with higher management levels, ensuring resource sharing, problem-solving, and the spread of successful strategies across the wider organization. Communication with frontline clinicians was an ongoing, intensive process involving regular updates, check-ins, and supportive reminders. Facility-level quality leaders were key to effective communication and performance tracking. Successful strategies were compiled in a shared playbook, creating a culture of implementing initiatives, practices, or lessons discussed during these meetings at the local facility level.

### Outcome measures

Our primary outcome was the CAUTI standardized infection ratio (SIR). Secondary outcomes included the IUC standardized utilization ratio (SUR) and the incidence of CAUTI per 1,000 catheter days. The monitoring of CAUTIs was carried out regularly by the infection prevention department using the conventional criteria set by the National Healthcare Safety Network (NHSN); the outcome measures were calculated using the standard NHSN methods.^10^

### Statistical methods

We evaluated the CAUTI SIR, IUC SUR, and CAUTI rates in the preintervention (P1, June 2020 to December 2021) and postintervention period (P2, January 2022 to June 2023). Additionally, we compared the CAUTI SIR to a preestablished benchmark (0.442, which represented the 30th percentile for the CAUTI SIR in 2020, selected to motivate an aspirational outcome). We analyzed the distribution of SUR and SIR data using the Shapiro-Wilk test and quantile–quantile (Q–Q) plots. Due to the non-normality in the SUR data, we used the Wilcoxon rank sum test to compare SUR values. On the other hand, the SIR and CAUTI rates results showed normal distributions, allowing us to use the one-sample *t* test. We set statistical significance at a *P* value of .05. However, computational constraints due to low to zero infection rates in three community hospitals limited statistical analysis at those sites. This approach allowed for robust statistical analysis despite the varied distribution patterns of the data.

## Results

### Alternatives to IUC

Policies, protocols, and products were refined to minimize IUC placement. The health system’s urinary management policies were updated, emphasizing their judicious use. A nursing practice guideline offered an algorithm for implementing bladder scans and straight catheterization, enabling the practical assessment and management of voiding difficulties. External male and female catheters became more accessible by expanding the selection of systemwide products and ensuring the availability of supplies in all units.

In intensive care units, where the need for urinary management is arguably the highest, the uptake of IUC-avoiding procedures was robust. Additional decision support tools were developed and disseminated to assist nursing staff in evaluating the necessity of IUC placement based on patient conditions. In intensive care units, straight catheterization was routinely considered as the primary option, minimizing the need for IUC placement.

Broad-scale campaigns were initiated across all facilities to decrease IUC use in operating room and emergency department settings. “Why the Foley” campaigns specifically targeted emergency departments, advocating for IUC use only under limited conditions. In other cases, patients were reassessed for potential IUC needs after admission.

### IUC insertion

The health system policy for adult urinary catheters was revised to incorporate a standardized protocol for urine sample collection from IUCs. A decision-making checklist and Infogram were introduced to help nursing staff ascertain when using an IUC was necessary. To foster standardization and promote best practices, prepackaged IUC kits and an insertion checklist were made readily available. Ongoing training programs focused on IUC insertion and maintenance reinforced caregivers’ knowledge, refined their skills, and enhanced their overall competencies.

### IUC maintenance

In 2022, a new protocol was instituted for chlorhexidine gluconate (CHG) bathing in patients with IUCs based on a comprehensive review of current evidence and an evaluation of its practicability within the UH Health System. Recognizing widespread staffing constraints, the CHG bathing protocol was designed to be seamlessly integrated into existing daily care routines. CHG bathing, already a practice for patients with indwelling devices, such as central venous catheters, was seen as an effective horizontal strategy that curtailed multiple types of infections. Initiatives to foster acceptance and understanding included comprehensive reviews of evidence-based practice, tailored staff training and educational programs, and the creation of a frequently asked question (FAQ) resource. The FAQ addressed common concerns and questions and used high-quality illustrations and supporting references, making it a user-friendly and highly effective tool for education and buy-in.

### IUC removal

System policies were revised to ensure the prompt removal of IUCs by mandating that nurses and providers evaluate and document their necessity daily, and by implementing a nurse-driven IUC removal protocol for qualified patients. A post-IUC removal voiding trial practice guideline was established to further promote patient voiding and the use of straight catheterization as an initial step before reinserting an IUC.

### Smart diagnostics

Practice guidelines and electronic medical record (EMR) order sets were put in place to ensure that IUCs in place for more than 48 hours were removed or replaced with a new catheter before sample collection. In addition, order sets replaced the separate ordering of urine cultures with urinalysis accompanied by culture only if indicated with few exceptions.

### Performance tracking

Utilizing real-time tracking systems, leaders were granted transparent visibility into patient care, while frontline caregivers could observe trends beyond their immediate patient assignments. Visual management boards served as a tangible and accessible tool for displaying relevant metrics, increasing overall visibility, and providing immediate context to these figures. CAUTI rates could be examined at a granular level in individual nursing units. Lists of patients using IUCs in each facility underwent daily reviews, and should documentation fail to justify its necessity, the nurse responsible for the patient was promptly contacted. The total number of days that each patient had an IUC received increased attention. This emerged as a critical data point, considering there is a 3%–5% increased risk of bacteriuria per catheter day.^11^

Nonetheless, challenges were encountered when accessing the desired data. Documentation of bladder scanning and straight catheterization protocols were not readily available with automated data; thus, we were not able to measure these utilization trends. A manual review of the EMR posed difficulties for consistent monitoring and real-time feedback. Accessing data from varying data systems in health system facilities proved to be challenging. At the nursing unit level, the daily use of dashboards was inconsistent. Despite these obstacles, data analytics remained a central focus in understanding and mitigating the incidence of CAUTIs.

The core CAUTI project team included representatives of infection prevention, quality, champions from nursing and medicine, and nursing education and supply chains. The nursing education team is a vital partner in crafting effective training programs and communication strategies. The supply chain team was indispensable for ensuring adequate and consistent stocking of essential equipment and products in every unit. Finally, when a diagnosis of CAUTI was made, strategies such as staff huddles were employed to provide a forum for immediate discussion and feedback among providers. This approach fosters collaboration, promotes understanding, and ensures a unified response to tackle CAUTIs.

### Outcomes

We evaluated the impact of targeted interventions on SUR, SIR, and CAUTI rates across 10 sites. The interventions led to various changes, as presented in Tables 2, 3, and 4. The analysis showed a mix of significant and nonsignificant changes from preintervention to postintervention periods.

**Table 2.** Indwelling urinary catheters (IUC) standardized utilization ratios (SUR) in periods 1 and 2

Hospital	Period 1 SUR (95% CI)	Period 2 SUR (95% CI)	*P* value
A	0.831 (0.824, 0.838)	0.811 (0.804, 0.817)	.283
B	0.694 (0.681, 0.708)	0.738 (0.725, 0.752)	.246
C	0.530 (0.517, 0.542)	0.618 (0.605, 0.631)	.012
D	0.661 (0.648, 0.674)	0.560 (0.547, 0.573)	.003
E	0.693 (0.676, 0.709)	0.738 (0.721, 0.756)	.102
F	0.819 (0.803, 0.835)	0.760 (0.744, 0.776)	.055
G	0.698 (0.670, 0.727)	0.915 (0.894, 0.937)	.001
H	0.296 (0.277, 0.316)	0.393 (0.371, 0.415)	.013
J	0.748 (0.711, 0.786)	0.960 (0.917, 1.004)	.013
K	0.689 (0.643, 0.737)	0.663 (0.613, 0.716)	.932

**Table 3.** Catheter-associated urinary tract infections (CAUTI) standardized infection ratios (SIR) in periods 1 and 2

Hospital	Period 1 SIR (95% CI)	Period 2 SIR (95% CI)	*P* value (Benchmark: .442)	*P* value (Benchmark: Pre-SIR)
A	0.75 (0.585, 0.948)	0.497 (0.365, 0.661)	.596	.023
B	0.339 (0.108, 0.817)	0 (0, 0.246)	.001	.007
C	0.38 (0.064, 1.256)	0.155 (0.008, 0.766)	.022	.066
D	0.842 (0.341, 1.751)	0.197 (0.01, 0.971)	.011	<.001
E	0.212 (0.011, 1.045)	0 (0, 0.646)	<.001	<.001
F	0.391 (0.099, 1.064)	0.288 (0.048, 0.95)	.198	<.001
G	0.654 (0.033, 3.223)	0 (0, 0.579)	.001	<.001
H	1 (NA, NA)	0 (NA, NA)	NA	NA
J	0 (0, 2.992)	0 (0, 2.462)	NA	NA
K	0 (NA, NA)	0 (NA, NA)	NA	NA

Note. CI, confidence interval.

**Table 4.** Catheter-associated urinary tract infection (CAUTI) rates in periods 1 and 2

Hospital	Period 1 CAUTI rates	Period 2 CAUTI rates	*P* value
A	1.235	0.806	.11
B	0.368	0	.004
C	0.279	0.112	.132
D	0.595	0.138	.001
E	0.148	0	.094
F	0.309	0.241	.545
G	0.137	0	.109
H	1.1	0	NA
J	0	0	NA
K	0	0	NA

Note. NA, not Applicable.

Table 2 presents the SUR values before and after the intervention using the Paired Wilcoxon rank sum test for comparison. Hospitals C, G, H, and J show statistically significant increases in their SURs, while hospital D shows a significant decrease; the remaining hospitals exhibit no significant changes.

Most hospitals showed a decrease in SIR from Period 1 to Period 2 (Table 3); hospitals B, C, D, E, and G showed significant improvements (*P* <.05) when compared to the .442 benchmark, indicating effective interventions. Hospitals A, B, D, E, F, and G also showed significant changes from their pre-SIR values, suggesting substantial reductions in infection rates. Hospitals H, J, and K had insufficient data for statistical comparison.

Table 4 shows the CAUTI rate per 1,000 catheter days. Hospital B and Hospital D showed statistically significant reductions in CAUTI rates. Hospitals A, C, E, F, and G showed decreases in CAUTI rates that were not statistically significant, while Hospitals J and K maintained zero CAUTI rates in both periods. Hospital H lacked sufficient data for statistical significance analysis.

## Discussion

This comprehensive, multifaceted QI approach highlights the potential for a systemic and sustainable reduction in CAUTI rates across a complex health system. By utilizing the FMS for implementing rigorous, evidence-based interventions and fostering a culture of accountability and collaborative problem-solving, significant strides were made toward transforming CAUTIs into a “never event.”

The success of infection prevention and control interventions in any hospital is largely influenced by the organizational culture; proven technical approaches to prevent infection have often faltered because of the failure of hospital staff to adopt them, and a cultural shift is necessary to address suboptimal infection prevention and control practices. A positive hospital culture—characterized by safety, leadership, innovation, and collaboration—can enhance adherence to infection prevention and control strategies, thereby reducing HAI rates. The importance of behavioral and socio-adaptive factors in change management for infection control initiatives has led to the development of the SHEA/IDSA/APIC Practice Recommendation on “Implementing strategies to prevent infections in acute-care settings,” focusing on bridging the gap between knowledge and practice. The authors discuss several key models, including the 4Es (engage, educate, execute, evaluate), the Behavior Change Wheel, and the Comprehensive Unit-based Safety Program, among others. These frameworks are designed to address behavioral and socio-adaptive challenges within healthcare environments, providing practical strategies to implement evidence-based interventions effectively. The practice recommendations emphasize the importance of understanding facilitators and barriers to change, as well as the critical role of continuous measurement and feedback in sustaining improvements, factors that are addressed in the model we described.^12^

The FMS’s emphasis on structured communication and real-time data analytics is the key strength of this approach, allowing for the generation of immediate, actionable insights into patient care trends. These insights, shared among teams, fostered a shared learning environment, promoting the dissemination of best practices and contributing to overall QI. A crucial aspect of this program was the intentional focus on minimizing the use of the IUC. By implementing strict criteria for IUC placement, promoting alternative urinary management techniques, and encouraging early IUC removal, the program directly addressed a critical modifiable risk factor for CAUTI. These measures, supported by policy revisions and data-driven feedback loops, ensured consistent and critical evaluation of IUC use. Improving diagnostic accuracy and promptness helps in effectively monitoring CAUTI rates and responding to potential outbreaks. However, challenges such as data access hurdles and inconsistent use of daily visual management boards must be addressed. Continued investment in data infrastructure and staff training is crucial to sustain improvements. The collaboration among nursing education, supply chain, and other specialized teams demonstrates the comprehensive nature of this approach.

In conclusion, our experience demonstrates that a systemic and data-driven approach underpinned by active collaboration and structured communication can effectively drive substantial improvements in CAUTI rates. By minimizing IUC use, promoting alternative urinary management techniques, and fostering a culture of data-informed, patient-centered care, CAUTIs are much rarer across the health system.
