# Reducing catheter-associated urinary tract infections in a large health system: a quality improvement approach using a fractal management system – ADDENDUM

**DOI:** 10.1017/ash.2025.23

**Published:** 2025-01-17

In the original publication of this article^1^, Lisa Bushong, MSN, RN, was omitted from the list of authors.

The article has been updated.
